# Long-Range PCR Amplification of DNA by DNA Polymerase III Holoenzyme from *Thermus thermophilus*

**DOI:** 10.1155/2015/837842

**Published:** 2015-01-19

**Authors:** Wendy Ribble, Shawn D. Kane, James M. Bullard

**Affiliations:** ^1^Replidyne, Inc., Louisville, CO, USA; ^2^Chemistry Department, The University of Texas-Pan American, SCNE 3.320, 1201 W. University Drive, Edinburg, TX 78541, USA

## Abstract

DNA replication in bacteria is accomplished by a multicomponent replicase, the DNA polymerase III holoenzyme (pol III HE). The three essential components of the pol III HE are the *α* polymerase, the *β* sliding clamp processivity factor, and the DnaX clamp-loader complex. We report here the assembly of the functional holoenzyme from *Thermus thermophilus* (*Tth*), an extreme thermophile. The minimal holoenzyme capable of DNA synthesis consists of *α*, *β* and DnaX (*τ* and *γ*), *δ* and *δ*′ components of the clamp-loader complex. The proteins were each cloned and expressed in a native form. Each component of the system was purified extensively. The minimum holoenzyme from these five purified subunits reassembled is sufficient for rapid and processive DNA synthesis. In an isolated form the *α* polymerase was found to be unstable at temperatures above 65°C. We were able to increase the thermostability of the pol III HE to 98°C by addition and optimization of various buffers and cosolvents. In the optimized buffer system we show that a replicative polymerase apparatus, *Tth* pol III HE, is capable of rapid amplification of regions of DNA up to 15,000 base pairs in PCR reactions.

## 1. Introduction

*Thermus thermophilus* (*Tth*) is an extreme thermophile which can grow at temperatures above 75°C [1]. Replication of the* Tth* genome is carried out by a multicomponent replicative polymerase similar to the well-characterized* E. coli* DNA polymerase III holoenzyme (pol III HE) [2, 3]. The replicative polymerases are composed of three major subassemblies: a specialized polymerase, a sliding clamp processivity factor, and a clamp-loading complex. The characteristics for replicative polymerases to replicate entire genomes relatively quickly are the speed of the polymerase and the fact that the polymerase is kept from dissociating from the DNA by virtue of being tethered to the substrate by association with the *β* clamp [4]. The *β* clamp forms a bracelet structure that encircles DNA and tethers a rapidly moving polymerase to the DNA [5]. The *β* subunit is efficiently loaded onto the double-stranded DNA in the presence of ATP by the DnaX complex. The DnaX complex is composed of the essential DnaX (*τ*/*γ*), *δ* and *δ*′, and two accessory proteins (*χ* and *ψ*) [6]. The minimal form of the* Tth* pol III HE, composed of *α*, *β*, DnaX, and *δ* and *δ*′, is capable of rapid and processive polymerization of DNA [7]. In previous work using NH_2_-terminal tagged forms of* Tth α*, *β*, DnaX, and *δ* and *δ*′, we have shown that the proteins composing the* Tth* pol III HE exhibit the same characteristics as observed for their* E. coli* homologs. Each of the subunits is absolutely required for DNA synthesis by the* Tth* pol III HE. The same protein-protein interactions occurring in the* E. coli* holoenzyme also take place in the* Tth* pol III HE. In the NH_2_-terminal-tagged form of* Tth *pol III HE the rate of synthesis was comparable to that of the* E. coli* holoenzyme. Overall the* Tth* pol III HE behaves as replicative polymerases from other systems [7].

The polymerase chain reaction (PCR) method of DNA amplification is a powerful and sensitive technique. It has broad applications in molecular biology, diagnostics, detection, identification, and forensic analysis [8]. Since it was originally described [9] and presented at Cold Spring Harbor [10], PCR techniques and reagents have undergone significant improvements. The first improvement involved the use of a heat-stable polymerase from* Thermus aquaticus* (*Taq*), which eliminated the need to replenish the reaction with fresh enzyme after each cycle [11]. This improvement allowed PCR reactions to move from a slow deliberate process to an automated fast moving process. The remaining major limitations to PCR are the yield of product, fidelity of the process, speed of the polymerase, and the size of the region of DNA that can be amplified. Attempts to increase the yield of PCR products include addition of helper proteins [12, 13], the use of additives [14, 15], and optimization of primers for increased yield from small amounts of starting material [16].

Improving fidelity with the goal of producing error-free PCR products has been a substantial challenge and has many bioscience companies still looking for a solution. This has, however, led to the discovery of a number of new thermostable enzymes that have error rates improved from the original* Taq* polymerase [17–19]. Yet with all these improvements the commercially marketed polymerases with the highest fidelity still misincorporate 1 base out of 16,000 up from 1 base out of 1600 seen with* Taq* polymerase [18]. Even with improved fidelity, the new high fidelity polymerases have the potential of one mistake in the amplification of every eight medium-sized bacterial genes.

Finally, efficiently amplifying very long regions of DNA has been a problem. Some approaches to improve amplification of long products include changing pH, using additives, decreasing denaturation times, increasing extension times, and addition of secondary polymerases [20–22]. Other approaches have been to create new proteins by conjugating to or incorporating a processivity factor into an existing polymerase [23–25]. However, most commercial PCR systems for amplification of long regions of DNA are a mixture, composed of a high level of nonproofreading polymerase such as* Taq* and smaller amounts of a proofreading enzyme [26]. The fact that many researchers are still striving to improve PCR systems indicates that a need remains for improved systems. Such a system would rapidly and reliably amplify long regions of DNA with high fidelity. We have exploited the properties of the multicomponent replicative polymerase that* Tth* uses to replicate its genome with the high fidelity required for genome stability to establish a system capable of high yield, high fidelity, and synthesis of long products.

Each component of the* Tth* pol III HE was purified as a native protein in order to avoid the problems that might arise by using proteins containing nonnative amino acid sequences as purification tags. We describe the purification of the subunits in their native form and show that the* Tth* pol III HE is rapid and processive in DNA replication. Under optimal conditions the* Tth* pol III HE is stable at temperatures to 98°C and remains active for the duration of the procedure. This is the first demonstration that a thermophilic replicative polymerase can function in PCR and amplify regions of DNA exceeding 15,000 bp.

## 2. Experimental Procedures

### 2.1. Construction of Expression Vectors

**Figure 1:**
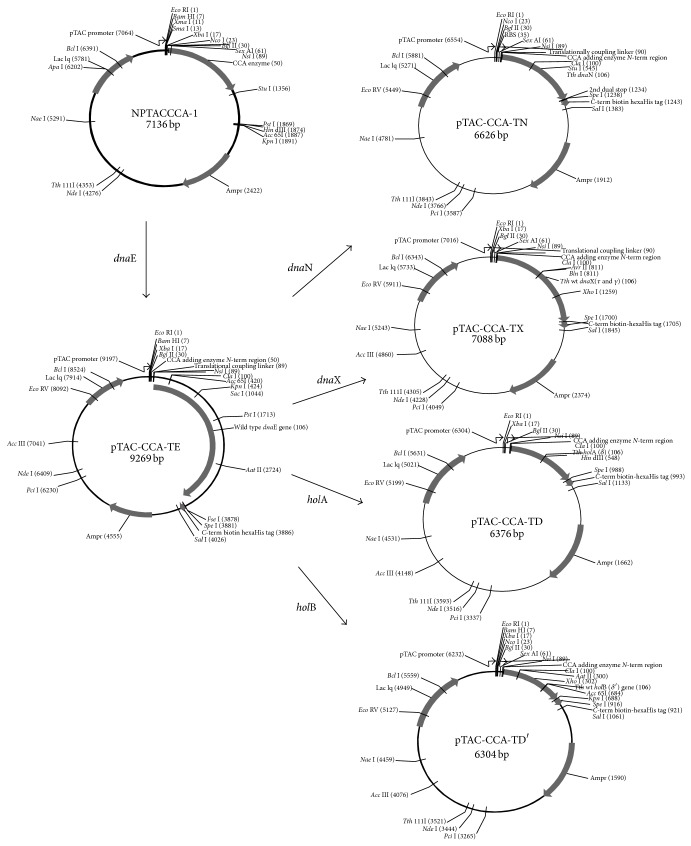
Constructs allowing* Tth* proteins expression by translationally coupling. Construction of plasmids expressing* Tth* proteins using translational coupling techniques is described under Section 2. The primers used in PCR of individual genes are listed in Table 1. The* dna*E gene (*α*) was first cloned as a translationally coupled construct (pTAC-CCA-TE). This construct was then used as a starting point in the construction of translationally coupled vectors containing* dna*B (*β*), DnaX (*τ*/*γ*),* hol*A (*δ*) and* hol*B (*δ*′), and* ssb* (SSB).

The gene encoding the tRNA nucleotidyl transferase (NT), the CCA adding enzyme, is contained within the NPTACCCA-1 plasmid (Figure 1) and is expressed at high levels (data not shown). To translationally couple the* Tth* genes to the NT gene nearly 98% of the gene encoding the tRNA NT was removed by cleavage with* Nsi*I and* Kpn*I. This resulted in only the 5′ 13 codons of the NT gene remaining. The* dna*E gene encoding* Tth α* was then translationally coupled to the remaining 5′ end of the tRNA NT gene in two steps. First, the 5′ end of the* dna*E gene was amplified by PCR. The sense primer (Table 1) added a sequence containing an* Nsi*I restriction site, a ribosome binding site (RBS), and eight downstream nucleotides (six form a* Cla*I site and the last two are the first two nucleotides of a TAA stop codon which overlap the A of the ATG start codon of the* dna*E gene). The antisense primer is complementary to a region downstream of a* Kpn*I restriction site in the* dna*E gene. The PCR product was cleaved with* Nsi*I and* Kpn*I and inserted between these restriction sites of NPTACCCA-1 creating the new plasmid pTAC-CCA-TEmp (not shown). This positioned the 5′ end of the* dna*E gene downstream and translationally coupled to the gene encoding tRNA NT but in a different reading frame. Second, the remainder of the* dna*E gene was removed from the plasmid pA1-NB-TE [7] by cleavage with* Kpn*I and* Sal*I and inserted between the* Kpn*I and* Sal*I restriction sites of pTAC-CCA-TEmp resulting in the pTAC-CCA-TE plasmid which contained the full length* dna*E gene (Figure 1).

The* dna*N gene encoding* Tth* pol III HE *β* subunit was amplified by PCR from plasmid pA1-NB-TN [7] using a sense primer containing a* Cla*I restriction site and the TA of a stop codon. This DNA sequence is in the same reading frame with the 5′ region of the CCA adding enzyme. The TA butts to the ATG start codon of the* dna*N gene forming the sequence TAATG. As with* dna*E, this places the* dna*N gene downstream and translationally coupled to the gene encoding tRNA NT but in a different reading frame. The antisense primer added a downstream* Spe*I restriction site. The PCR product was inserted between the* Cla*I and* Spe*I restriction sites of pTAC-CCA-TE. This replaced the* dna*E gene with the* dna*N gene. The resulting plasmid was named pTAC-CCA-TN (Figure 1). The genes encoding* Tth* DnaX (*dna*X), *δ* (*hol*A), *δ*′ (*hol*B), and SSB (*ssb*) were cloned into the pTAC-CCA-TE plasmid by the same process, resulting in pTAC-CCA-TX, pTAC-CCA-TD, pTAC-CCA-TD′, and pTAC-CCA-TSSB, respectively (Figure 1). The primers used for PCR of each of the genes are shown in Table 1.

### 2.2. Cell Growth and Preparation of Fraction I

Expression vectors were transformed into* E. coli* AP1.L1 (F-,* omp*T* hsd*SB(rB-) (srl-recA)306::Tn10, T1 phage-resistant isolate). Cells were grown at 37°C in F broth (yeast extract 14 g/L; tryptone 8 g/L; K_2_HPO_4_ 12 g/L; KH_2_PO_4_ 1.2 g/L; glucose 1%) plus 100 *μ*g/mL ampicillin to an OD_600_ of 0.6–0.8 and expression was induced by addition of isopropyl *β*-D-1-thiogalactopyranoside (IPTG) to 1 mM. Cells were harvested at 3 hours after induction and were resuspended in an equal volume of Tris-sucrose buffer (50 mM Tris-HCl (pH 7.5), 10% sucrose) and quick frozen in liquid nitrogen.

Cells were lysed and the recovered supernatant constituted Fraction I (Fr I) [27]. For the initial purification step, the ammonium sulfate (AS) concentration in which >80% of the* Tth* target protein precipitated out of solution was determined for each* Tth* pol III subunit. The protein pellets resulting from AS precipitation of the large-scale Fr I solutions were quick frozen in liquid nitrogen and stored at −80°C.

### 2.3. Gel Electrophoresis, Protein Analysis, and Reagents

SDS-PAGE analysis was performed using either 12% or 4–12% acrylamide precast gels (Novex NuPAGE; Invitrogen) with MOPS running buffer (Invitrogen). Benchmark unstained protein molecular weight markers were used (Invitrogen). Protein concentrations were determined by the method of Bradford [28] using bovine serum albumin as a standard. Oligonucleotides were from Integrated DNA Technologies (Coralville, IA). All other chemicals were obtained from either Sigma Aldrich (St. Louis, MO) or Fisher Scientific (Pittsburg, PA). Radioactive isotopes were from PerkinElmer (Waltham, MA).

### 2.4. Purification of* Tth α*

The 35% saturated AS precipitated protein pellets containing* Tth α* was dissolved in Buffer A (50 mM Tris-HCl, (pH 7.5), 25% glycerol, 1 mM EDTA, and 1 mM DTT) and clarified by centrifugation (16,000 ×g) yielding Fraction II (Fr II). Fr II was further purified using a Butyl Sepharose Fast Flow (Pharmacia Biotech) column equilibrated using Buffer A plus 1 M AS. The column (5.5 × 13 cm) was poured using 70% of the butyl resin. The remaining 30% of butyl resin was mixed with Fr II. To this mixture, 1 volume of saturated AS was added slowly while stirring over a period of 1 hour. This mixture was then added to the column and washed with Buffer A plus 1 M AS. The protein was eluted in a linear gradient beginning with Buffer A plus 1 M AS and ending in Buffer A plus 50 mM KCl. Fractions were pooled and the protein precipitated by addition of AS to 50% saturation. Protein pellets were resuspended in Buffer B (20 mM HEPES, (pH 7.5), 10% glycerol, 0.1 mM EDTA, 5.0 mM DTT) and further purified using a Sephacryl S200 HR (Pharmacia Biotech) gel filtration column equilibrated in Buffer B.

### 2.5. Purification of* Tth β*

The 40% saturated AS precipitated protein pellets containing* Tth β* were resuspended in Buffer C (50 mM Tris-HCl (pH 7.5), 10% glycerol, 0.5 mM EDTA, and 5 mM DTT) and clarified by centrifugation (16,000 ×g) (Fr II). The sample was heated to 65°C for 30 min and the precipitated protein removed by centrifugation (16,000 ×g). The soluble fraction was further purified using Q Sepharose (Pharmacia Biotech) equilibrated in Buffer C plus 50 mM NaCl. The protein was eluted in Buffer C containing a 150–300 mM NaCl linear gradient. The fractions were pooled and the proteins precipitated by addition of AS to 50% saturation. The *β* subunit was further purified using Butyl Sepharose resin as described for *α*.

### 2.6. Purification of* Tth* DnaX (*τ*/*γ*)

The 35% saturated AS precipitated protein pellets containing* Tth* DnaX were resuspended in Buffer C, and the sample was clarified by centrifugation (16,000 ×g) (Fr II). Fr II was heated to 65°C for 30 min and precipitated proteins were removed by centrifugation (16,000 ×g). The DnaX was purified using an SP Sepharose column equilibrated in Buffer C plus 50 mM NaCl and eluted in Buffer C containing a 50–300 mM NaCl linear gradient.

### 2.7. Purification of* Tth δ*

The 45% saturated AS precipitated protein pellets containing* Tth δ* were resuspended in Buffer D (25 mM Tris-HCl (pH 7.5), 10% glycerol, 1.0 mM EDTA, and 1 mM DTT) and clarified by centrifugation (16,000 ×g) (Fr II). A Q Sepharose High Performance (Amersham Pharmacia) chromatography column equilibrated in Buffer D plus 10 mM KCl was used in the first purification step.* Tth δ* eluted in the column flow-through fraction. This pool (Fr III) was further purified using Macro Prep Methyl HIC Support (BioRad) column chromatography. The methyl resin was equilibrated in Buffer C plus 1 M ammonium sulfate. The column was poured using 60% of the resin. The remaining 40% of resin was mixed with Fr III. To this mixture, 1 volume of saturated AS was added slowly while stirring over a period of 1 hour. This mixture was added to the column and the flow-through fraction was collected by gravity. The column was washed with Buffer C plus 1 M AS and the proteins were eluted in Buffer C containing a 0.9 to 0.1 M linear reverse gradient of AS.* Tth δ* was further purified using a Sephacryl S300 HR (Pharmacia Biotech) gel filtration column equilibrated in Buffer E (50 mM Tris-HCl, (pH 7.5), 20% glycerol, 100 mM NaCl, 1 mM EDTA, and 5 mM DTT) yielding Fr V.

### 2.8. Purification of* Tth δ*′

The 45% saturated AS precipitated protein pellets containing* Tth δ*′ were resuspended in Buffer A and purified using Butyl Sepharose Fast Flow (Pharmacia Biotech) resin as described above (Fr III).* Tth δ*′ was further purified using Octyl Sepharose Fast Flow (Pharmacia Biotech) column chromatography. The octyl resin was equilibrated using Buffer C plus 0.5 M AS. The column was poured using 70% of the octyl resin. The remaining 30% of octyl resin was mixed with Fr III. To this mixture, 0.5 volume of saturated AS was added slowly while stirring over a 1 hour period. This mixture was added to the column and washed with Buffer C plus 200 mM AS.* Tth δ*′ was eluted in the wash and was collected in fractions (Fr IV).* Tth δ*′ was further purified using a Sephacryl S300 HR (Pharmacia Biotech) gel filtration column equilibrated using Buffer E.

### 2.9. Optimization of the Thermostability of* Tth* Pol III Holoenzyme

The initial buffer used in M13gori assays contained 50 mM Hepes, (pH 7.5), 20% glycerol, 5 mM magnesium acetate (MgOAC), 1 mM ATP, and 50 mM potassium glutamate [7]. To increase the temperature at which* Tth *pol III HE retained activity, assays were developed (50 *μ*L) in which the master mix (25 *μ*L) (containing different buffers (at varying pH), concentrations of glycerol, MgOAC, and ATP) were mixed with 19 *μ*L of 0.22 *μ*g (0.004 *μ*M) M13mp18 (New England Biolabs), 0.02 *μ*M M13 primer (5′-GGGTAACGCCAGGGTTTTCCCAGTCACGAC-3′), 0.05 *μ*M *δ*′, 0.01 *μ*M *δ*, 1.4 *μ*M *β* (monomer), 0.05 *μ*M DnaX (monomer), and 0.5 *μ*M *α* and cycled at various temperatures before adding 6 *μ*L dNTP to start the reaction. Assays consisted of 5 cycles of melting temperature for 20 s, reannealing temperature (60°C) for 2 min, and extension temperature (70°C) for 2 min to mimic PCR cycles. The melting temperature was initially at 85°C, but as optimization of a component increased the thermostability of the holoenzyme this temperature was increased. DNA synthesis was initiated by addition of dNTP mix (40 *μ*M dATP, dGTP, dCTP, and 18 *μ*M [^3^H]dTTP (100 cpm/pmol)) and incubation was continued at 70°C for 2 min. The reaction was terminated by addition of 3 mL 10% TCA. The solution was filtered under vacuum through Whatman GF/C glass microfiber filters and the radioactivity retained was measured as described [7].

To test additives and develop an optimized buffer system to further increase thermostability of the holoenzyme, 16 *μ*L of the optimized basic buffer (20 mM TAPS-Tris (pH 7.5), 15% glycerol, 15 mM MgOAc, and 1 mM ATP) and 6 *μ*L of test component in various concentrations were mixed and cycled as described above. The reactions were initiated by addition of dNTP mix and continued as described above.

### 2.10. Assay to Determine DNA Synthesis in PCR-Like Reactions

Assays (25 *μ*L) contained the optimized buffer system (the optimized basic buffer plus: 10% sorbitol (or 15% maltitol), 1% PEG 20000, and 1 M trimethylamine N-oxide (TMAO)) and 0.22 *μ*g (0.004 *μ*M) M13mp18, 0.06 *μ*M primer and 250 *μ*M dATP, dGTP, dCTP, and 112 *μ*M [^3^H]dTTP (20 cpm/pmol). Enzyme concentrations were the same as used in the thermostability optimization. The reaction was allowed to proceed through 20 cycles composed of 94°C/30 s, 60°C/1 min, and 70°C/2 min. Individual reactions were stopped after every other cycle to the end of the 20 cycles. The assays were carried out in duplicate, one for gel analysis and one for quantification of DNA synthesis. For gel analysis each 25 *μ*L reaction was loaded into a well of a 0.7% agarose gel (15 × 15 cm) and electrophoresis was for 45–90 min at 100 V using a subcell GT apparatus (BioRad). Gels were stained for 5 min in TAE buffer containing 5 *μ*g/mL ethidium bromide. The gels were destained for 15 min in TAE buffer and DNA bands visualized using a Kodak Image Station 440.

### 2.11. PCR Reactions

PCR reactions (25 *μ*L) using pET Blue-2 plasmid (Novagen) as a template contained the optimized buffer system plus 200 *μ*M each dNTP, 2 *μ*M each primer, and 0.05 *μ*g plasmid. Enzyme concentrations were the same as used in the thermostability optimization. PCR cycles consisted of 94°C/30 s, 55°C/1 min, and 72°C/2 min. Following cycling, the samples were incubated at 72°C for an additional 5 min. PCR reactions were analyzed using 0.7% agarose gel electrophoresis as described above. Reactions using* Taq* DNA polymerase (18038-018, Invitrogen) for comparison were carried out as per the manufacturer's conditions. The* Tth* holoenzyme and* Taq* polymerase reactions contained the same amount of primer and template in the same reaction volume. PCR reactions (50 *μ*L) using Lambda DNA-*Hind*III Digest (New England BioLabs) as a template contained double the amount of enzyme.

## 3. Results

### 3.1. Cloning of* Tth* Proteins

We have previously expressed all of the proteins in NH_2_ terminal His-tagged forms [7]. However, difficulties in expressing* Tth* proteins as native proteins lead us to hypothesize that since the* Tth* genome is high in G/C content, the mRNA may have the potential to form secondary structure near the 5′ end of the gene that does not affect translation in* Tth* at elevated temperatures, but that greatly diminishes expression carried out in* E. coli* at 37°C. To overcome this problem, we designed a translationally coupled expression system. The plasmid NPTACCCA-1 contains the gene expressing tRNA nucleotidyl transferase (NT) under control of the pTAC promoter (Figure 1). In this system the tRNA NT is expressed in* E. coli* at high levels (to 50% of total cellular proteins). To translationally couple the genes encoding the* Tth* proteins to the gene encoding the tRNA NT, most of the tRNA NT gene was removed, leaving only 13 codons of the 5′ end of the gene. A sequence containing an RBS followed by a TAA stop codon was added in the same reading frame as the gene encoding the tRNA NT. In similar constructs, genes encoding* Tth α*, DnaX, *δ*, *δ*′, and *β* were added in such a way that the start codon of each gene overlapped the second A residue of the stop codon. This coupled the* Tth* gene to the sequence encoding the tRNA NT 5′ by virtue of the new RBS, but in a different reading frame. All of the* Tth* genes were overexpressed in this system to 5–20% of total cellular protein.

### 3.2. Purification and Activity of Pol III Holoenzyme Proteins

**Figure 2:**
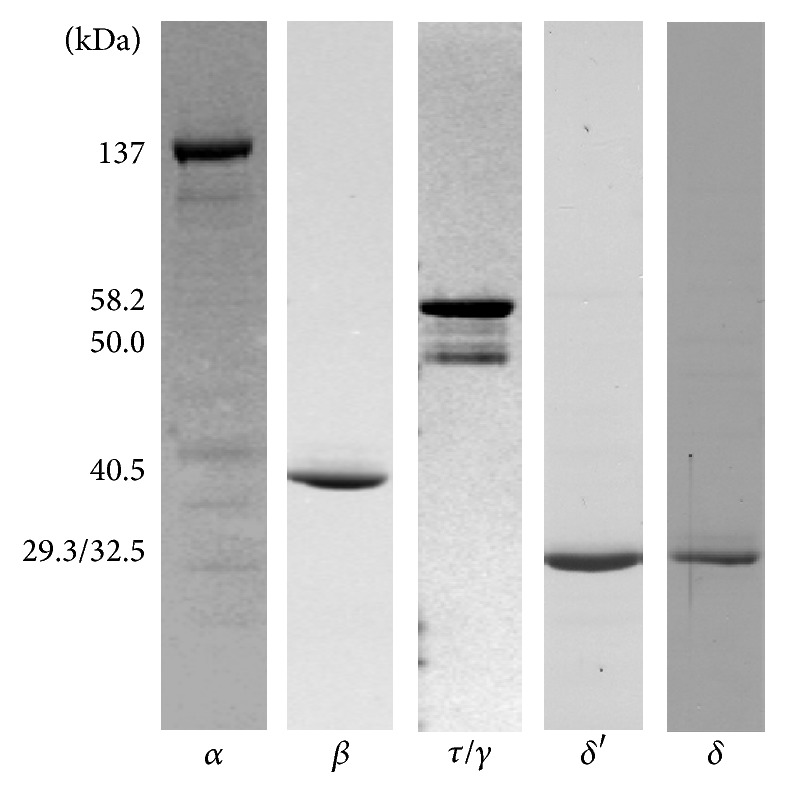
Purification of* Tth* DNA pol III holoenzyme subunits. *α*, *β*, *τ*/*γ*, *δ*, and *δ*′ were purified to at least 95% homogeneity as described under Section 2.

Native forms of the subunits comprising the* Tth* pol III HE were expressed and purified as described in “Section 2” using a variety of purification schemes (Table 2) to at least 95% homogeneity by visual examination (Figure 2). Electrophoresis of the *τ* and *γ* proteins revealed minor bands migrating between the two subunits. However, western blot analysis using monoclonal antibodies against the DnaX protein indicated that the minor bands between *τ* and *γ* were degradation products of *τ* (data not shown). The *γ* form of DnaX also appeared to resolve as a doublet. Previous work indicated that even though the *τ* and *γ* forms of DnaX are encoded by the same gene, in* Tth* the mRNA encoding *γ* is distinct from that encoding *τ*, as a result of transcriptional slippage [29]. This work also indicated that two forms of *γ* were present. The smaller form of *γ* results from a −1 slippage bringing a UGA stop codon located two codons downstream into the same reading frame as the* dna*X gene. The larger form of *γ* is a result of a −2 slippage which brings a UGA stop codon located sixteen codons downstream into the same reading frame as the* dna*X gene. The* dna*X gene in* Tth* also contains a translational frame-shift signature [30] which allows the expression of *γ* from a single mRNA encoding both *τ* and *γ* by a translation frame-shifting mechanism. Which mechanism is occurring during overexpression of* Tth* DnaX in* E. coli* cells is not clear.

**Figure 3:**
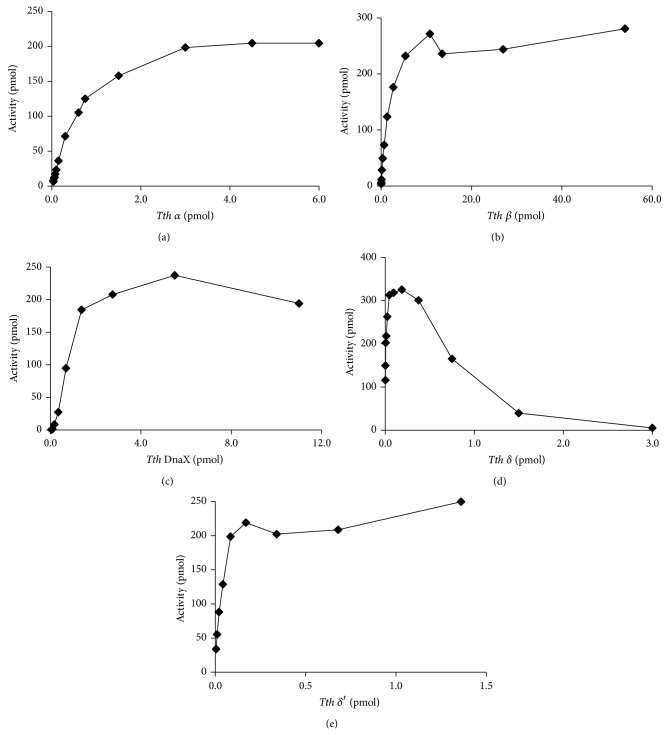
Requirements for individual components of the* Tth* pol III holoenzyme. DNA synthesis was used to monitor the activity of the subunits [7]. The pol III holoenzyme was reconstituted using native forms of each subunit. The components that were not being tested were held constant at saturating concentrations (0.05 *μ*M *δ*′, 0.01 *μ*M *δ*, 1.4 *μ*M *β* (monomer), 0.05 *μ*M DnaX (monomer), and 0.5 *μ*M *α*) while the test subunit was titrated into the reactions as indicated. Reactions were at 55°C. The subunits titrated into the reactions were (a) *α*, (b) *β*, (c) DnaX, (d) *δ*, and (e) *δ*′. The concentrations of proteins were calculated based on the molecular mass of single subunits. “Activity” is defined as the total pmol of nucleotides incorporated.

Using NH_2_-terminal His-tagged forms of *α*, *β*, *τ*/*γ*, *δ*, and *δ*′, we previously showed that each subunit was required for processive DNA synthesis [7]. Each of the native subunits of the* Tth* pol III HE purified here was titrated into identical assays to determine optimal activity for downstream assays (Figure 3). Each subunit was required for synthesis of DNA complementary to ssM13 DNA. The *δ* subunit stimulated an increase in activity to a maximum at 0.2 pmol of *δ*, but at greater concentrations of *δ* the DNA synthesis activity decreases (Figure 3(d)). When the native form of *δ* was titrated into assays in which the system contained all native subunits or in assays containing all His-tagged forms of the subunits the same results were seen. Alternatively, when the native form of *δ* was replaced with the His-tagged from of *δ* there was no decrease in activity observed (data not shown). This is likely due to the fact that *δ* acts to load and unload the *β* subunit onto the DNA [32, 33] and at high concentrations of *δ* (greater than the DnaX complex), the *β* subunit is preferentially unloaded, thereby causing a decrease in the processivity of the holoenzyme. It is possible that the NH_2_-terminal His-tag may interfere with the optimal ability of *δ* to unload *β*.

### 3.3. Stabilization of* Tth* Pol III Holoenzyme at High Temperatures

**Figure 4:**
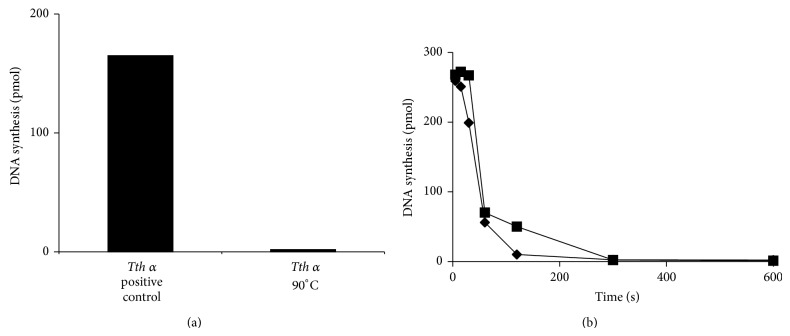
Thermostability of* Tth α*. Gap-filling polymerase assays [31] were used to determine loss of activity of* Tth α* at elevated temperatures. (a) The enzyme mix was heated to 90°C for 2 min and then combined with the substrate mix and incubated for an additional 5 min at 60°C. The positive control was not subjected to the heat challenge step. (b) Components of the complete* Tth* holoenzyme were mixed at room temperature and incubated at 80°C (■) or 85°C (*◆*). Samples were removed at the indicated times.

**Figure 5:**
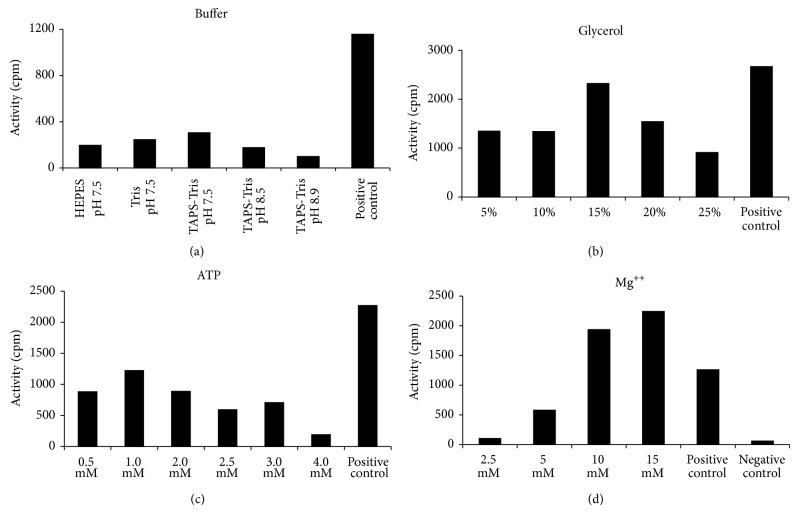
Assay to optimize thermostability of the* Tth* pol III holoenzyme. Assays were as described under Section 2. Positive controls are reactions carried out using initial buffer at 55°C. (a) Effect of different buffers in reactions on activity at 85°C. HEPES, Tris-HCl, and TAPS-Tris buffer concentrations were 25, 50, and 20 mM, respectively. (b) Assays containing different percent of glycerol tested at 87°C. (c) Assays containing the indicated concentrations of ATP were tested at 88°C. (d) Assays containing the indicated concentrations of magnesium acetate (Mg^++^) were tested at 88°C. Negative control is minus Mg^++^.

The *α* subunit has proven to be an Achilles heel for the* Tth* pol III HE to be active at increased temperatures. As an initial purification step, fractions containing *α* lost activity when heated to 65°C. To ascertain the heat stability, purified *α* was heated to 90°C for 2 min and 100% loss of activity was observed (Figure 4(a)). Under these conditions, *α* lost approximately 80% of activity at 80 or 85°C within 30 sec (Figure 4(b)). For use in PCR,* Tth* pol III HE must retain activity across multiple cycles of at least 94°C, so various reaction components and conditions were tested to identify optimal conditions. The melting temperature for each cycle was set at 85°C. Initially, assays were carried out in the presence of various buffers: Tris-HCl, HEPES, TAPS-Tris, TAPS-Bis-Tris, TAPS-KOH, HEPES-Bis-Tris, HEPES-Bis-Tris Propane, and TAPS-Bis-Tris Propane. Reactions were carried out using each buffer at various pH settings (Figure 5(a)). The condition promoting the highest polymerase activity at 85°C was 20 mM TAPS-Tris (pH 7.5). Optimal conditions for the other assay components were then determined to be 15% glycerol, 1 mM ATP, and 15 mM MgOAc (Figures 5(b), 5(c), and 5(d)). Under the new conditions contained in the optimized basic buffer, the* Tth* pol III HE retained activity to 91°C over the course of the reaction. During the thermal stabilization studies, assays were conducted in the presence or absence of* Tth* SSB and no difference in activity was observed. Therefore, SSB was not included in later thermal stabilization or PCR reactions.

**Figure 6:**
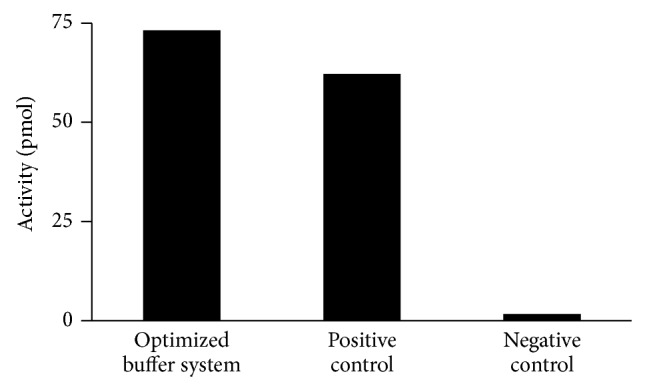
A comparison of the activity of* Tth* pol III holoenzyme at 55°C versus 98°C. Assays containing the optimized buffer system were cycled 5 times at 98°C/20 s, 60°C/2 min, and 70°C/2 min prior to addition of the dNTP mix and then incubation was continued at 70°C for an additional 2 min. Positive control contained the original buffer mix and was cycled as for optimized buffer system reactions but all cycle temperatures were at 55°C. Negative controls were cycled as for the optimized buffer system reactions but contained the original buffer mix.

Next, 59 different additives including cosolvents, sugars, crowding agents, detergents, betaines, salts, and metals were tested (Table 3). As one additive was observed to increase thermostability it was incorporated into the assay mix and subsequent assays were carried out at an increased temperature. Using this method an optimized buffer system was developed which contained 20 mM TAPS-Tris (pH 7.5), 15% glycerol, 15 mM MgOAc, 1 mM ATP, 10% sorbitol (or 15% maltitol), 1% PEG 20000, and 1 M TMAO. In this system, the thermostability of* Tth* pol III HE was increased so that activity was stable at 98°C for the length of the reaction (Figure 6).

### 3.4. DNA Synthesis by* Tth* Pol III Holoenzyme in PCR-Like Reactions

**Figure 7:**
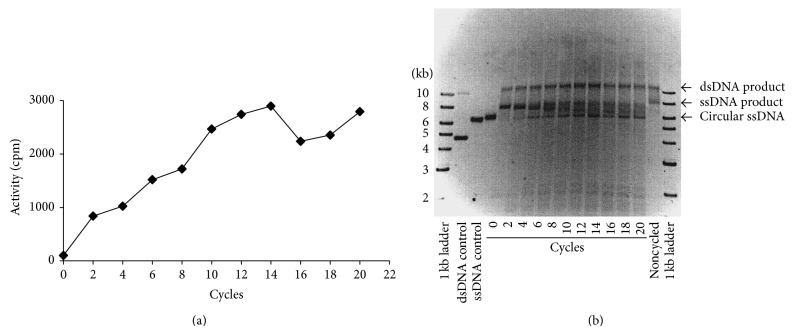
Determination of the thermostability of* Tth* pol III HE in cycled reactions. (a) The effect of cycle number on the amount of DNA synthesized. (b) Products from the asymmetric PCR-like reactions. The circular ssM13mp18 DNA served as the starting material. 0 cycles refers to the mix that was not cycled at any temperature. All other lanes contain products from reactions cycled for the indicated times. Each cycle consisted of steps at 94°C/30 s, 60°C/1 min, and 70°C/2 min. The noncycled reaction contains primed M13mp18 template plus* Tth* pol III HE incubated at 70°C for 2 min.

We tested whether the* Tth* pol III HE could synthesize long DNA products during each cycle and for how many cycles this ability was retained. Using the optimized buffer system, we monitored the ability of the* Tth* pol III HE to synthesize long regions of DNA in PCR-like cycles. The template was circular single-stranded M13mp18 and the primer was a 30-nucleotide oligomer complementary to the template. The primer-template ratio was 15 : 1 and the reaction was allowed to proceed through 20 cycles composed of 94°C/30 s, 60°C/1 min, and 70°C/2 min. Two sets of assays were carried out in duplicate and reactions were stopped after every other cycle. The product from one reaction was analyzed for DNA synthesis by scintillation counting and the product from the duplicate reaction was analyzed by gel electrophoresis. An increase in activity was observed through 14 cycles and then remained constant; because of the 15 : 1 primer-template ratio at this point all the primer may have been exhausted (Figure 7(a)). The samples used for gel analysis allowed determination of the length of DNA synthesized during each cycle and the relative amounts of full-length product (Figure 7(b)). In Figure 7(b), the double-strand DNA (dsDNA) control contained both super-coiled double-stranded M13mp18 (lower band) and nicked double-stranded M13mp18 (less intense upper band). The ssDNA control contained circular single-stranded M13mp18 DNA. Since this was an asymmetric PCR-like assay, there was only one strand of the substrate amplified and unlike normal PCR, in which two primers are used, the amount of substrate did not double with each cycle but remained static. The top band (dsDNA product) is clearly the replicated circular ssDNA (now nicked dsDNA) that represents the annealed product and the original template. This band remained at approximately the same intensity because the double-stranded product from the previous cycle would have been denatured releasing the circular ssDNA template for use in the next cycle. The bottom band (circular ssDNA) is the circular M13mp18 template. The central band (ssDNA product) is the linear product from replication of the circular M13mp18 and migrates slower on the gel as a result of linearity. The graph in Figure 7(a) indicated more DNA was being synthesized; however the ssDNA product in the gel did not seem to increase beyond cycle 6. This was an artifact of the use of ethidium bromide to identify single-stranded DNA. The band representing the new ssDNA product was not quantitative since ethidium bromide interacts with ssDNA inefficiently and cannot be used to quantify single-stranded DNA. Since the circular single-stranded DNA contains supercoiling it interacts with ethidium bromide in a more quantitative manner. The noncycled reaction contained the same reaction mix as the cycled reaction, except the primer was preannealed to the template. This reaction was not cycled but was incubated at 70°C for 2 min and then stopped. This reaction resulted in a band containing nicked full-length dsDNA and a band containing apparently a partially synthesized dsDNA. There was no ssDNA substrate remaining, indicating that there was complete conversion of ssDNA to dsDNA. In the lane containing uncycled (zero) reaction, only circular ssDNA template was observed. The slight difference in the position of the ssDNA in the uncycled lane as compared to the ssDNA control may be due to a buffer-induced upward shift. Since there was no cycling, no annealing of primer to template was possible and therefore no DNA was synthesized. In cycle #2 there was no remaining circular ssDNA, but the circular ssDNA appeared in increasing amounts as the cycle number increased, indicating that all ssDNA was not being primed in later cycles. The linear ssDNA product (7249 bp) appears more intense in cycle #2; this is actually the overlap with another replicative product which appears to be migrating lower with every cycle. We do not know what this additional replicative product represents; perhaps this represents incompletely synthesized products or more likely it is circular single-stranded template with different levels of supercoiling. The results obtained from this experiment indicated that the* Tth* pol III HE remained stable in PCR-like cycles and was capable in each cycle of synthesizing DNA to over 7000 bp within 2 min.

### 3.5. *Tth* Pol III Holoenzyme in PCR Reactions

**Figure 8:**
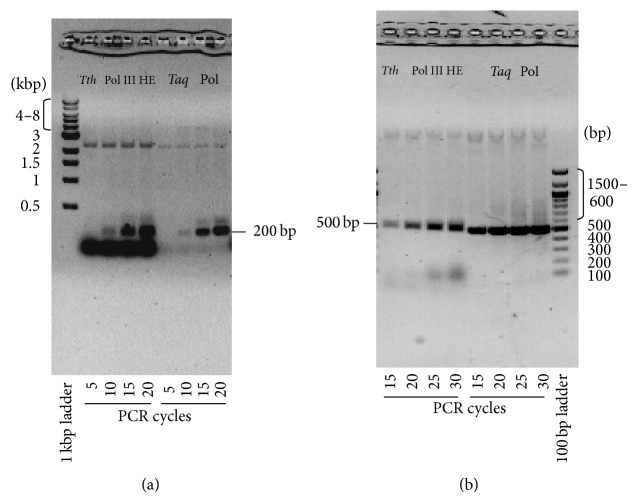
PCR reactions for amplification of short regions of DNA. PCR reactions and agarose gel analysis were as described under Section 2. PCR cycles consisted of 94°C/30 s, 55°C/1 min, and 72°C/2 min.* Taq* Pol indicates PCR reactions using* Taq* DNA polymerase (18038-018, Invitrogen) per manufacturer's instructions. Primers used are shown in Table 3. (a) PCR reactions using primers designed to yield a 200 bp PCR product. (b) PCR reactions using primers designed to yield a 512 bp PCR product. The number of PCR cycles is indicated at bottom of the figures.

To determine if the* Tth* pol III HE could perform a PCR reaction, the activities of* Tth* pol III HE and* Taq* polymerase were compared. The substrate for amplification of DNA in the initial PCR reactions was pET Blue-2 plasmid (Novagen). The primers (Table 4) were designed to yield a PCR product of 200 bp. PCR reactions were carried out as described under “Section 2” and contained either* Tth* pol III HE or* Taq* polymerase and the reactions were performed for 5, 10, 15, and 20 cycles. The* Tth* pol III HE amplified the 200 bp DNA as well as the* Taq* polymerase did at each cycling time (Figure 8(a)). There appear to be shadow bands in the* Tth* holoenzyme reactions that are not obvious in the* Taq* polymerase reactions. We believe that this is a result of the interaction of the* Tth* pol III HE with this particular primer set because when different primers were used this was not observed.

**Figure 9:**
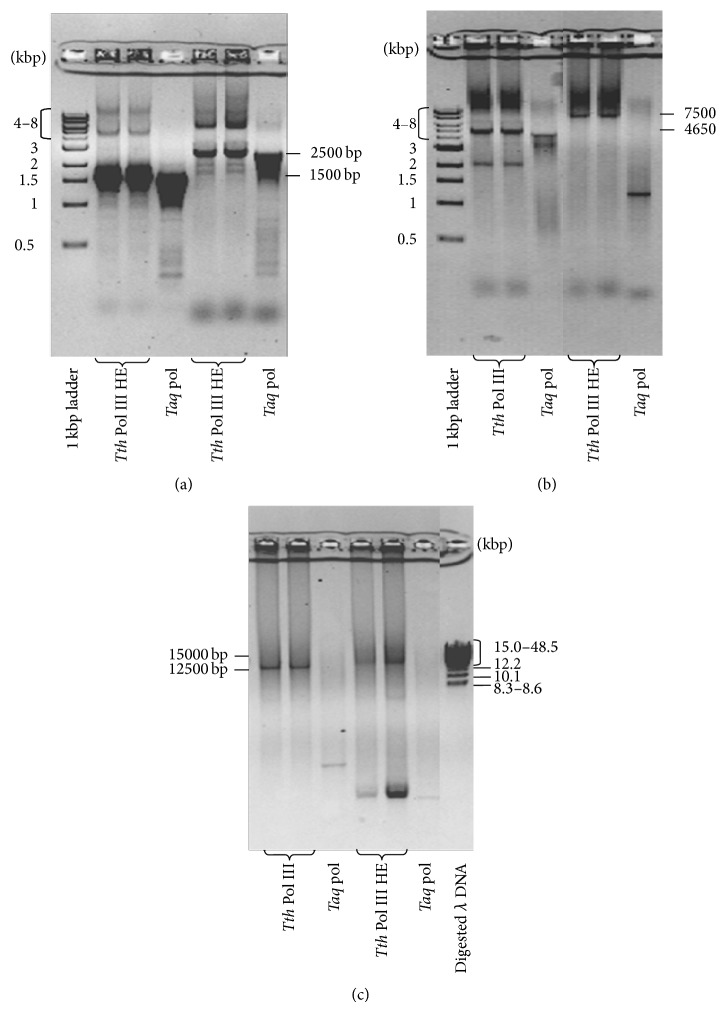
PCR reactions to amplify intermediate and long regions of DNA. Reactions were conducted as described under Section 2. PCR reactions were allowed to proceed for 30 cycles. The same amount of primer and template was used in* Tth* pol III HE and* Taq* polymerase reactions. Primers used are shown in Table 3. PCR product sizes are as indicated. (a) The template was pET Blue-2 plasmid (Novagen) and primers were designed to yield 1500 and 2500 bp products. (b) The template was Lambda DNA-*Hind*III Digest and primers were designed to yield a 4650 and 7500 bp product. (c) The template was Lambda DNA-*Hind*III Digest and primers were designed to yield a 12500 and 15000 bp product.

Next, we determined the efficiency of the* Tth* holoenzyme in amplification of longer regions of DNA. Primers were selected that would yield a 500 bp product. The number of cycles in the reactions was changed to 15, 20, 25, and 30 cycles. In each of the reactions the amplification of the DNA by* Tth* pol III HE was equal to or only slightly less than that observed with* Taq* polymerase (Figure 8(b)). These assays also indicated that* Tth* pol III HE continued to have higher yields to at least 30 cycles. Next, primers were designed to yield PCR products that were 1500 and 2500 bp in length in 30-cycle assays (Figure 9(a)). In these reactions, the yield of PCR products from reactions containing* Tth* pol III HE was equal to that in reactions containing* Taq* polymerase. The* Tth* pol III HE PCR reactions contained more distinct full-length DNA products and fewer abortive DNA fragments than contained in the* Taq* PCR products.

To analyze the ability of* Tth* pol III HE to amplify longer regions of DNA, *λ* genomic DNA was used as the template. In PCR reactions designed to yield products approximately 5000 bp in length, the* Tth* pol III HE reactions were observed to contain at least 2-fold more PCR product than contained in the* Taq* polymerase reactions (Figure 9(b)). When the length of the DNA to be amplified was increased to 7500 bp, there was a distinct full-length PCR product observed in the reactions containing* Tth* pol III HE, but at this length,* Taq* polymerase was no longer able to produce a full-length PCR product (Figure 9(b)). It appears that* Taq* polymerase is not fast or processive enough to complete a full-length 7500 bp PCR product within the 2 min extension time. Finally, primers were designed to amplify 12500 and 15000 bp regions of the *λ* DNA. The PCR reactions containing* Tth* pol III HE yielded full-length products while no PCR products were observed in the* Taq* polymerase reactions (Figure 9(c)). The ability of* Tth* pol III HE to perform long-range PCR in a short time is consistent with its DNA synthesis rate of over 350 bp/sec determined using M13 substrates [7].

## 4. Discussion

Replication of cellular genomes is carried out by a specialized replicative DNA polymerase that is highly processive. In bacteria studied to date, this apparatus is composed of three functional components: the catalytic polymerase, a sliding clamp processivity factor, and a clamp-loading multiprotein complex. We have expressed, purified, and assembled this apparatus in its native form from the thermophilic organism* Thermus thermophilus*. We have shown that all of the functional components required in Gram-negative [3] and Gram-positive [34] mesophilic bacteria are also required in the* Tth* apparatus.* Aquifex aeolicus*, another thermophilic organism, also has been shown to contain a replicative polymerase with these functional components [35]. These findings suggest that all bacteria may utilize a similar mechanism in the replication of their genomes. We have shown previously that the same protein-protein interactions observed between the components of the replicative polymerase in the mesophilic bacteria also occur in* Tth* [7]. In this same work,* Tth* pol III HE was shown to polymerize the entire M13Gori template in one binding event, indicating that the replicase is highly processive. The entire 7.2 kb M13mp18 template was replicated within 20 seconds, yielding a DNA synthesis rate of at least 360 nt/s [7], which is similar to the rate of synthesis for replicative polymerases from mesophilic bacteria [34, 36].

The* Thermus* sp. is composed of extreme thermophiles which grow at temperatures greater than 75°C and in these conditions are able to synthesize thermostable proteins [37]. A higher proportion of charged amino acids at the expense of polar or noncharged amino acids, especially at the surface of proteins, appears to be at least partially responsible for the stability of these proteins [38]. Since* Tth α* was partially inactivated at temperatures above 65°C and completely inactivated at 80°C within 30 sec (Figure 4), this presented a problem for use of* Tth* pol III HE in PCR. An increase in thermostability was achieved through the optimization of the buffer, Mg^++^, ATP, and glycerol concentrations. This approach allowed partial retention of activity by* Tth* pol III HE to near 91°C.

Previous work has identified conditions that would allow increased stabilization of individual enzymes from both mesophilic and thermophilic origins. Certain cosolvents have been shown to enhance the thermostability of xylanase from* Thermomonospora* sp. [39] and* Thermotoga* sp. [40], alpha-chymotrypsin [41], and papain [42]. The stability of bovine pancreatic trypsin and Moloney murine leukemia virus reverse transcriptase were increased in the presence of different sugars [43, 44]. Detergents were used to increase the stability of a DNA polymerase purified from* Thermotoga* sp. at higher temperatures [45]. Betaines [46, 47] or the addition of chemical chaperones such as trimethylamine* N*-oxide (TMAO) improved the yield of active proteins and addition of metals was shown to stabilize the structure of misfolded proteins [48, 49]. These findings led us to test an extensive list of additives for their effects on the thermostability of* Tth* pol III HE. By iteratively testing additives and combinations of additives, we were able to increase the stability of the* Tth* pol III HE so that full activity was retained to 98°C for at least five PCR-like cycles and for 30-cycle PCR reactions in which 94°C was used as a heat step. To determine if the activity could be retained for the denaturation steps required for PCR, the* Tth* pol III HE was compared with* Taq* polymerase initially using primer sets that yielded short PCR products. The length of the region of DNA to be amplified was increased incrementally to determine how well* Tth* pol III HE performed compared to* Taq*. In these reactions the* Tth* pol III HE PCR products were comparable to that of* Taq* polymerase but contained fewer abortive product fragments.

Since PCR was first developed, the production or longer PCR product has been an ongoing goal and continues today [50–53]. Since* Tth* pol III HE is able to synthesize the entire* Tth* genome in one binding event, we were encouraged to test whether it would be capable of long-range PCR (greater than 5000 bp). PCR reactions using *λ* DNA as a template were designed to determine if* Tth* pol III HE could produce long-range PCR products, and we showed that amplification of DNA regions up to 15,000 bp was possible.* Taq* polymerase produced no products in comparable reactions. The observation that* Taq* polymerase could not amplify DNA regions above 5000 bp highlights the importance of speed of polymerization for amplification of long regions of DNA.* Taq* polymerase in the presence of a secondary polymerase is able to amplify much longer regions of DNA (up to 35 kbp). To accomplish this, extension times have to be extended to as much as 26 min [21, 26]. This would increase the time to do a 30-cycle PCR reaction to as long as 15 hours. This suggests that an on/off switching time during the elongation step may be rate limiting and indicates a clear advantage of a single high fidelity, high processive polymerase.

This is the first time that a replicative multicomponent polymerase has been shown to have the ability to perform PCR. The advent of a single-enzyme system that is capable of amplifying long regions of DNA in a short period of time opens up exciting possibilities. Aside from the ability to do longer-range PCR the other major limitations of enzymes in use today remain to be, even though increased from that of* Taq* polymerase, a lack of a high level of fidelity seen with replicative polymerases [18].* Taq* polymerase with no added proofreading function misincorporates nucleotides at a rate of 0.01 to 0.27 × 10^−6^ [54, 55], while higher-fidelity polymerases have been developed that are 10 times more accurate [18, 56]. However, the accuracy of the replicative polymerase from* E. coli*, pol III HE,* in vitro*, is approximately 700 times higher than that of* Taq* polymerase [57]. The current system lacks the proofreading subunit, epsilon, that contains a 3′ > 5′ exonuclease function and accounts for enhanced fidelity. In the* E. coli* system, base selection by the polymerase accounts for up to 10,000-fold higher fidelity than does the proofreading function of epsilon and the addition of epsilon only decreases the error rate by 3- to 5-fold [57, 58]. Therefore, the addition of epsilon to this system would modestly increase the fidelity but would likely have adverse effects on the rate of polymerization. In summary, the ability to amplify very long DNA regions efficiently and accurately could potentially be a useful addition to the list of improvements to PCR.

## Figures and Tables

**Table 1:** Primers used for PCR amplification of genes encoding *Tth* pol III holoenzyme subunits.

Gene amplified	Primer direction	Sequence (5′-3′)
*dna*E5′	Sense	GGATATGCATTGAGGAGGATCGATTAATGGGCCGCAAACTCCGC
*dna*E5′	Antisense	CGGCTCGCCAGGCGCACCAGG
*dna*N	Sense	ACTGATCGATTAATGAACATAACGGTTCCCAAG
*dna*N	Antisense	GACTACTAGTCTACTAGACCCTGAGGGGCACCACC
*dna*X	Sense	ACTGATCGATTAATGAGCGCCCTCTACCGCCGC
*dna*X	Antisense	GACTACTAGTTTATTATATACCAGTACCCCCTATC
*hol*A	Sense	ACTGATCGATTAATGGTCATCGCCTTCACCGGGG
*hol*A	Antisense	GACTACTAGTCATCAACGGGCGAGGCGGAGG
*hol*B	Sense	ACTGATCGATTAATGGCTCTACACCCGGCTCACCC
*hol*B	Antisense	GACTACTAGTCATCATGTCTCTAAGTCTAAGGCC
*ssb *	Sense	ACTGATCGATTAATGGCTCGAGGCCTGAACCGCG
*ssb *	Antisense	GACTACTAGTCATCAAAACGGCAAATCCTCCTCC

**Table 2:** Purification summary for the *Tth* pol III holoenzyme subunits.

Protein	Starting material (g)	Fraction	Total protein (mg)	Total volume (mL)	Total activity (units^a^)	Specific activity (U/mg)
*α*	400	Fr I (cell lysate)	30420	1600		
		Fr II (35% AS)^b^	850	200	1.1 × 10^8^	0.15 × 10^6^
		Fr III (butyl pool)	71	300	1.1 × 10^8^	1.6 × 10^6^
		Fr IV (S-200 pool)^c^	10	7.5	2.2 × 10^7^	2.3 × 10^6^
		FR IV (reassay)^d^	10	7.5	8.0 × 10^6^	0.8 × 10^6^

*β*	200	Fr I (cell lysate)	8780	760		
		Fr II (40% AS)	1000	200	9.4 × 10^7^	0.1 × 10^6^
		Fr II (heat shock)	150	380	8.1 × 10^7^	0.5 × 10^6^
		Fr III (Q pool)	50	60	3.2 × 10^7^	0.67 × 10^6^
		Fr IV (butyl pool)	23	20	1.7 × 10^7^	0.75 × 10^6^

DnaX	300	Fr I (cell lysate)	24500	1140		
		Fr II (35% AS)	1258	250	5.0 × 10^8^	0.4 × 10^6^
		Fr II (heat shock)	523	600	5.0 × 10^8^	0.9 × 10^6^
		Fr III (SP pool)	200	440	2.8 × 10^8^	1.4 × 10^6^

*δ*	300	Fr I (cell lysate)	15300	930		
		Fr II (45% AS)	1850	2400	3.4 × 10^9^	1.8 × 10^6^
		Fr III (Q pool)	115	2471	3.6 × 10^9^	32.0 × 10^6^
		Fr IV (methyl pool)	14	100	1.7 × 10^9^	123.0 × 10^6^
		Fr V (GF pool)	5	24	5.8 × 10^9^	122 × 10^6^

*δ*′	200	Fr I (cell lysate)	19000	1400		
		Fr II (45% AS)	4000	540	1.5 × 10^10^	3.5 × 10^6^
		Fr III (butyl pool)	90	970	1.0 × 10^10^	15.5 × 10^6^
		Fr IV (octyl pool)	15	200	1.5 × 10^9^	90 × 10^6^
		Fr V (GF pool)	4.1	54	8.2 × 10^8^	200 × 10^6^

^a^One unit of activity is 1 pmol of total deoxyribonucleotide incorporated per min.

^b^The initial assays in *α* purification used a gap-filling assay to monitor nonprocessive polymerase activity [31].

^c^One-fourth of Fr III was used to prepare Fr IV.

^d^ *Tth* *α* Fr IV was reassayed in the reconstitution assay described in Experimental Procedures.

**Table 3:** Summary of additives tested to increase the thermostability of *Tth* pol III holoenzyme.

Cosolvents	Sugars	Crowding agents	Detergents
Glycerol (5–25%)^a^ Sorbitol (5–25%) Mannitol (2.5–10%) Maltitol (5–20%) 1-Methyl-pyrrolidinone (5–20%) 1-Methylindole (5–20%) 2-Pyrrolidinone (5–20%) Acetamide (0.25–1 M) Trimethylamine N-oxide (10 mM–1 M) Tertiary butane (5–20%) Trimethyl ammonium chloride (50 mM–1 M) Methylsulfonylmethane (0.12–6%)	Trehalose (140–720 mM) Sucrose (60 mM–1.2 M) *β*-Cyclodextrin (0.72–7.2 mM) *α*-Cyclodextrin (0.84–8.4 mM) Glucose (3–21%) D-Fructose (5–20%) D-Mannose (5–20%) D-Galactose (2–20%) Arabinose (5–80 mM)	PEG 400 (1–5%) PEG 4000 (2–5%) PEG 8000 (2–5%) PEG 20000 (2–5%) CM cellulose (0.16–1.2%) Polyvinylpyrrolidone (0.01–3%) Polyvinyl alcohol (0.5–4%) Ficol (0.5–3%)	Tween 20^b^ NP-40 Pluronic acid Zwittergent 3-08 Zwittergent 3-10 Zwittergent 3-12 Zwittergent 3-14 Zwittergent 3-16 Chaps Chaps SO N-Octyl-sucrose Caprolyl Sulfobetaine Myristyl-sulfobetaine SB 3-10 SB 3-14 N-Octyl-*β*-glucopyranoside N-Octyl-*β*-D-thioglucopyranoside

Betaines	Salts	Metals	Other

NDSB 195 (50 mM–1 M) NDSB 201 (50 mM–1 M) NDSB 256 (0.5 mM–1 M) 3-1-Pyridino-1-propan-sulfonate (50 mM–1 M) Betaine monohydrate (0.25–2 M) Betaine hydrochloride (0.25–1.25 M)	Potassium glutamate (25–200 mM) Sodium acetate (25–200 mM) Sodium citrate (25–200 mM)	Zinc sulfate (0.25–2 *μ*M) Magnesium sulfate (0.5–4 *μ*M)	L-Proline (0.12–1.2 M) Ethylene glycol tetraacetic acid (EGTA) (5–25 mM)

^a^Values in parenthesis represent ranges of concentrations tested.

^b^Detergents were tested at two concentrations, at CMC and at 5X lower than CMC.

**Table 4:** Primers used in *Tth* pol III holoenzyme PCR reactions.

Template DNA	Primer direction	Sequence (5′-3′)	PCR product (bp)
pET Blue-2	Sense	TAATACGACTCACTATAGGG	200
pET Blue-2	Antisense	GTCGTTTTACAACGTCGTGA	
pET Blue-2	Sense	TAATACGACTCACTATAGGG	516
pET Blue-2	Antisense	GCTAACGCAGTCAGGAGTATT	
pET Blue-2	Sense	CAATACTCCTGACTGCGTTA	1500
pET Blue-2	Antisense	GAATGAAGCCATACCAAACGA	
pET Blue-2	Sense	CAATACTCCTGACTGCGTTA	2000
pET Blue-2	Antisense	CACGCTGTAGGTATCTCAGTT	
*λ* DNA	Sense	CCGTTCTTCTTCGTCATAA	4650
*λ* DNA	Antisense	GATGCCGTTCATGACCTGATAA	
*λ* DNA	Sense	CCGTTCTTCTTCGTCATAA	7500
*λ* DNA	Antisense	GCAGCACAAATGCCACAGGTTCAT	
*λ* DNA	Sense	CCGTTCTTCTTCGTCATAA	12500
*λ* DNA	Antisense	CCATATTCTGTGCAATACCAT	
*λ* DNA	Sense	CCGTTCTTCTTCGTCATAA	15000
*λ* DNA	Antisense	CAGGCAGAGTCTCATGTAACT	

## Abbreviations

*Tth*: * Thermus thermophilus*
Pol III: DNA polymerase III (*α*)
*Tth* pol III HE: * Tth* DNA polymerase III holoenzyme
DnaX complex: A complex containing DnaX (*τ*/*γ*), *δ* and *δ*′
RFII: Replicative form II
*Taq*: * Thermus aquaticus* polymerase I
RBS: Ribosome binding site
PEG: Polyethylene glycol
PVA: Polyvinyl alcohol
PVP: Polyvinylpyrrolidone
EGTA: Ethylene glycol-bis-(2-aminoethylether)
NDSB: Nondetergent sulfobetaine
CMC: Critical micelle concentration.
